# Neuroendocrine Mechanisms of Acupuncture in the Treatment of Hypertension

**DOI:** 10.1155/2012/878673

**Published:** 2011-12-18

**Authors:** Wei Zhou, John C. Longhurst

**Affiliations:** ^1^Department of Anesthesiology, David Geffen School of Medicine, University of California Los Angeles, Los Angeles, CA 90095, USA; ^2^Department of Medicine, University of California Irvine, Irvine, CA 92697, USA

## Abstract

Hypertension affects approximately 1 billion individuals worldwide. Pharmacological therapy has not been perfected and often is associated with adverse side effects. Acupuncture is used as an adjunctive treatment for a number of cardiovascular diseases like hypertension. It has long been established that the two major contributors to systemic hypertension are the intrarenal renin-angiotensin system and chronic activation of the sympathetic nervous system. Recent evidence indicates that in some models of cardiovascular disease, blockade of AT1 receptors in the rostral ventrolateral medulla (rVLM) reduces sympathetic nerve activity and blood pressure, suggesting that overactivity of the angiotensin system in this nucleus may play a role in the maintenance of hypertension. Our experimental studies have shown that electroacupuncture stimulation activates neurons in the arcuate nucleus, ventrolateral gray, and nucleus raphe to inhibit the neural activity in the rVLM in a model of visceral reflex stimulation-induced hypertension. This paper will discuss current knowledge of the effects of acupuncture on central nervous system and how they contribute to regulation of acupuncture on the endocrine system to provide a perspective on the future of treatment of hypertension with this ancient technique.

## 1. Introduction

Hypertension affects approximately 1 billion individuals worldwide [1]. Hypertension is the most common chronic disorder in the United States, affecting 29% of the adult population [1]. The prevalence of this disorder increases with age; for normotensive middle-aged adults in the US, the lifetime risk of developing hypertension approaches 90% [2]. Although a number of treatment strategies have been developed for this disease, treatment has not been perfected and often is associated with adverse side effects.

Hypertension is the final outcome of a complex interaction between genetic and environment factors that act on physiological systems involved in blood pressure (BP) regulation (i.e., those that influence intravascular fluid volume, myocardial contractility and vascular tone) [3]. Evidence suggests that increased sympathetic neural activity plays a role in causing hypertension in some subjects who have a genetic tendency toward increased sympathetic activity as a result of repetitive psychogenic stress, obesity, or high sodium intake [3]. An important hypothesis in the pathogenesis of essential hypertension involves an interaction between high dietary sodium intake and defects in renal sodium excretion, which can be influenced by sympathetic neural activity and the renin-angiotensin-aldosterone system [3]. Enhanced sympathetic activity increases the secretion of renin and angiotensin. Angiotensin II enhances renal tubular sodium reabsorption directly and indirectly through increased release of aldosterone.

Acupuncture increasingly is being accepted as an alternative medical therapy in the United States. Manual acupuncture and its potent alternative, electroacupuncture (EA), have been used in Asia to treat a number of cardiovascular diseases including hypertension. Many Western physicians, however, are reluctant to recommend acupuncture, because its action in the treatment of hypertension remains controversial and because the physiological mechanisms determining its actions are largely unknown. This paper will discuss current knowledge of the effects of acupuncture on central nervous system and how they contribute to regulation by acupuncture of the endocrine system to provide a perspective on the future of treatment of hypertension with this ancient technique.

## 2. Clinical Study of Acupuncture in Treatment of Hypertension

**Figure 1:**
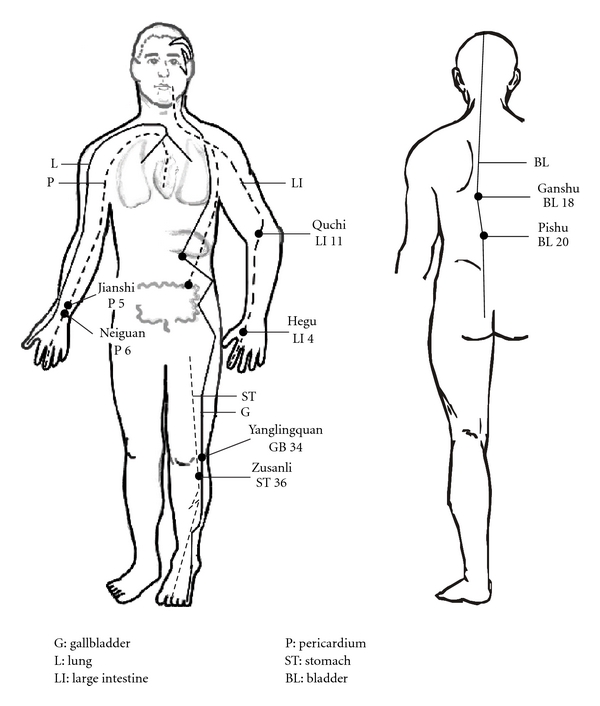
Location of acupoints along meridians. Note that although all meridians are bilateral, they are only drawn on one side for simplicity. Abbreviations of meridians: G: gallbladder; L: lung; LI: large intestine; P: pericardium; ST: stomach; BL: bladder.

In the past three decades, there have been a number of clinical studies focused on the effectiveness of acupuncture at specific acupoints to reduce BP in essential hypertension. As early as the 1950s, publications in China reported that acupuncture effectively reduced BP in hypertensive patients [4, 5]. In 1975, Tam found that acupuncture produced a significant reduction in systolic and diastolic BP in 24 out of 28 patients with essential hypertension [6].  Figure 1 shows a number of acupoints found to be effective in reducing BP, including pericardium 5, 6 (P 5, 6), stomach 36 (ST 36), large intestine 4, 11 (LI 4, 11), bladder 18, 20 (BL 18, 20), and gallbladder 34 (GB 34) [7, 8].

## 3. Acupoints Selection

We have evaluated the point specificity in EA treatment of reflex-induced hypertension caused by the gallbladder or splanchnic nerve (SN) stimulation in cats. This visceral reflex leads to stimulation of the sympathetic nervous system through the activation of cardiovascular premotor sympathetic neurons in the rostral ventrolateral medulla (rVLM). We observed that EA at P 5-6 (pericardial meridian, overlying the median nerve) and LI 10-11 (large intestine meridian, overlying the deep radial nerve) are most effective in reducing reflex-induced hypertension. EA at LI 4–L7 (large intestine and lung meridians, overlying branches of median and the superficial radial nerve) and ST 36-37 (stomach meridian overlying the deep peroneal nerve) are less effective, while EA at LI 6-7 and K1-B67 does not influence BP. Furthermore, direct stimulation of the deep or superficial nerves underneath the acupoints produces similar results [9, 10]. Similar observations have been made in a rat model employing gastric distension to elevate BP [11, 12].

## 4. Stimulation Parameters

EA rather than manual acupuncture has been used in many studies on cardiovascular related diseases, because the parameters of EA can be precisely controlled so the results are reproducible, whereas the outcome from manual acupuncture is operator dependent and therefore, is not as reproducible. A low frequency of 2 Hz is used more frequently to treat hypertension, because EA induces frequency-dependent release of neuropeptides. EA at 2 Hz produces a significant increase in enkephalin-like immunoreactivity but not in dynorphin immunoreactivity, whereas 100 Hz increases dynorphin immunoreactivity but not enkephalin immunoreactivity [13]. The similar results were confirmed in humans [14]. In the brain, enkephalins and endorphins as well as their associated *δ*- and *μ*-opioid receptors have been shown to be more important in modulating the cardiovascular actions of EA than dynorphin (*κ*-opioid) [15].

In our rat model of reflex hypertension, sham acupuncture involving needle insertion without manipulation at P 5-6 or LI 6-7 acupoints did not attenuate the gastric distention-induced hypertension, thus demonstrating that this procedure can serve as a control for EA. However, EA at P 5-6, H 6-7 (overlying the ulnar nerve) or ST 36-37 with low current (2 mA) and low frequency (2 Hz) for 30 min inhibited the reflex-induced hypertension. Increasing the stimulation frequency to 40 or 100 Hz did not inhibit the elevated BP. In this regard, we observed a reciprocal relationship between the frequency of stimulation and the afferent response. Thus, it appears that low-frequency, low-current EA in a point-specific manner optimally influences reflex-induced hypertension [11, 12].

## 5. Central Regulation of Blood Pressure

An increasing number of studies have demonstrated a critical role for the central nervous system in the development and maintenance of hypertension. In particular, increases in sympathetic nerve activity and alterations in arterial baroreflex function appear to contribute to the pathogenesis of this disease [16]. The development of hypertension in various animal models of hypertension, such as the spontaneously hypertensive rat (SHR), the renin transgenic (TGR mRen2) rat, the Dahl salt-sensitive rat, and the deoxycorticosterone acetate- (DOCA-) salt rat, is associated with increases in sympathetic activity. Increased sympathetic nerve activity elevates BP through arteriolar constriction and by increasing the force and rate of contraction of the heart to increase cardiac output. Renal sympathetic nerve activity also stimulates renin secretion that activates the systemic renin-angiotensin system leading to angiotensin (Ang) II-induced vasoconstriction and sodium retention [17]. Alteration of arterial baroreflex function has also been implicated in the development of hypertension [18]. Carotid sinus and aortic arch baroreceptors respond to changes in BP by modulating parasympathetic and sympathetic outflow and, hence, heart rate, cardiac output, and vascular tone. In response to a static increase in BP, the baroreflex resets towards a higher pressure [19]. In hypertensive conditions, resetting of the operational point of the arterial baroreflex, therefore, contributes to maintaining increased BP rather than opposing it. Similar to animal models of hypertension, hypertension in human subjects is associated with increases in sympathetic activity and blunted arterial baroreflexes [3, 18, 20, 21].

In hypertensive animals, functional changes within the central nervous system have been detected largely in hypothalamic and medullary areas that modulate sympathetic outflow [22]. Ang II contributes to cardiovascular regulation via its action at various hypothalamic and medullary areas to enhance sympathetic outflow, blunt the sensitivity of the baroreflex, and stimulate secretion of vasopressin [23, 24].

Over the past decade, we have examined the central neural regulation of visceral reflex-induced hypertension by acupuncture in different regions of brain, including the rVLM, hypothalamic arcuate nucleus, midbrain ventrolateral periaqueductal gray (vlPAG) nuclei, medullary nucleus raphé pallidus (NRP), and dorsal horn and intermediolateral column of the spinal cord.

## 6. EA Inhibition of Neural Activity in the rVLM

The rVLM plays a critical role in the regulation of BP [25]. Inhibition of neuronal function in this nucleus results in large decreases in BP [26]. We have demonstrated previously that both low-frequency electro- and manual acupuncture inhibit elevated BP as well as premotor sympathetic neural firing in the rVLM [12]. Administration of naloxone (nonspecific opioid receptor antagonist) or gabazine (*γ*-aminobutyric acid or GABA type A receptor blocker) in the rVLM abolishes the EA modulation [27]. The rVLM is an important brain stem region that processes somatic inputs during acupuncture stimulation. Electrophysiological studies of neuronal activity in the rVLM have shown that as compared to cardiovascular inactive points (LI 6-7, G 37–39), P 5-6 and certain acupoints along the large intestine meridian (LI 4–11), located over deep somatic neural pathways (median and deep radial nerves), provide more afferent input to cardiovascular premotor sympathetic neurons in the rVLM [10]. This observation likely explains why acupuncture over these deep nerves most effectively lower elevated sympathetic outflow and BP.

## 7. Neurotransmitters in rVLM, Arcuate, and vlPAG

Early studies in several models of hypertension suggested that EA lowers the elevated BP through the release of opioids, GABA, nociceptin, and serotonin (or 5-hydroxytryptamine, 5-HT) in the rVLM [28–32]. More recently, we have demonstrated that the EA inhibition of visceral reflex-induced hypertension in cats is related to the activation of *μ*- and *δ*-, but not *κ*-opioid receptors in the rVLM, suggesting that endorphins, enkephalins, and perhaps endomorphin, but not dynorphin, are mainly responsible for EA modulation of cardiovascular responses. 

Immunohistochemical staining studies have demonstrated the presence of enkephalinergic neurons in the rVLM and endorphinergic neurons in the arcuate nucleus that project directly to the rVLM and that both neurotransmitter systems are activated by EA [33]. EA inhibits the reflex hypertension through opioid-mediated inhibition of glutamate in the rVLM [34]. Electrophysiological studies [24] have shown that reciprocal excitatory glutamatergic (NMDA and non-NMDA) projections exist between the arcuate nucleus and vlPAG that may participate in the EA inhibition of cardiovascular function. This reciprocal projection may include a cholinergic component in the arcuate nucleus but not in the vlPAG [35].

Furthermore, EA, through presynaptic endocannabinoid CB1 receptor stimulation, reduces the vlPAG release of GABA but not glutamate during EA [36]. Reduced GABA disinhibits vlPAG neurons, thus increasing their activity, which, in turn, through an action in the NRP inhibits rVLM cardiovascular sympathetic neurons and related sympathoexcitatory reflex responses [37]. It is clear, therefore, that a variety of neurotransmitter systems underlie the cardiovascular modulation of EA. This includes both excitatory and inhibitory neurotransmitters, with their importance varying between the different nuclei.

## 8. Long-Loop Pathway for EA Cardiovascular Modulation

The role of the hypothalamic arcuate nucleus and its interaction with the vlPAG and rVLM in the EA-cardiovascular sympathoexcitatory responses has been extensively studied [10, 31, 38, 39]. Microinjection of the excitatory amino acid DLH, into the arcuate nucleus augments the responses of vlPAG neurons, while microinjection of small volumes (50 nL) of kainic acid (KA) causes reversible depolarization blockade that transiently deactivates arcuate neurons and decreases the vlPAG responses to SN stimulation [31]. Additionally, EA increases SN-evoked discharge of vlPAG neurons, a response that can be blocked by microinjection of KA into the arcuate nucleus. Microinjection of DLH into the arcuate nucleus, like EA, inhibits the reflex increase in BP induced by application of bradykinin to gallbladder for approximately 30 min. Finally, microinjection of KA into the arcuate blocks the inhibitory influence of EA on the reflex hypertension. As such, these results suggest that excitatory projections from the arcuate nucleus to the vlPAG appear to be essential to the inhibitory influence of EA on the reflex increase in BP induced by SN and gallbladder afferent stimulation.

## 9. vlPAG-rVLM Projections

The vlPAG provides inhibitory input to premotor sympathetic neurons in the rVLM to ultimately reduce sympathetic outflow and reflex elevations in BP [39]. Direct axonal projections from the vlPAG to the medulla have been documented in tract tracing studies [40]. However, a vlPAG projection to the raphé, in particular the nucleus raphé obscurus (NRO) also exists and has been thought to form an indirect pathway from the vlPAG to the rVLM that is involved in the EA-cardiovascular response [41]. Recent studies have suggested, however, that the NRP, located more ventrally than the NRO or the nucleus raphé magnus, contains more cells activated during median nerve stimulation with EA at the P 5-6 acupoints, as judged by the concentration of c-Fos labeling [42]. Chemical blockade of the NRP with KA or kenurenic acid transiently reverses activation of neurons in the rVLM during stimulation of the vlPAG as well as EA modulation of visceral excitatory reflexes [43]. Furthermore, the NRP inhibits rVLM activity, including activity of bulbospinal premotor sympathetic neurons. Serotonin projections from the raphé acting on 5-HT_1A_ receptors in the rVLM complete the vlPAG-NRP-rVLM circuit to modulate cardiovascular activity [43]. Thus, an indirect connection from the vlPAG to the rVLM involving a serotonergic connection between the NRP and the rVLM plays an important role in the long-loop modulation of cardiovascular sympathetic outflow during reflex visceral stimulation. These studies do not eliminate the possibility that direct projections between the vlPAG and the rVLM also might serve a functional role in EA-cardiovascular modulation. The direct or indirect projections from the vlPAG to the rVLM complete the long-loop pathway and provide an important source for the inhibitory influence of EA on rVLM premotor neurons and ultimately sympathoexcitatory cardiovascular responses [41].

## 10. Arcuate rVLM Projections

As noted previously, neurons in the vlPAG receive convergent input from a number of somatic nerves stimulated during EA as well as from the arcuate nucleus. Bilateral microinjection of KA into the rostral vlPAG partially reverses rVLM neuronal responses and cardiovascular inhibition during DLH stimulation of the arcuate. Conversely, depolarization blockade of the caudal vlPAG completely reverses arcuate evoked rVLM responses [41]. In parallel studies, we have observed that arcuate neurons can be antidromically activated from the rVLM and that arcuate perikarya are labeled with a retrograde tracer microinjected into the rVLM [41]. Many neurons from the arcuate that project to the rVLM are activated by EA stimulation (c-Fos positive) and they frequently contain opioid peptides, particularly *β*-endorphin [44]. As such, the vlPAG, particularly the caudal vlPAG, appears to be required for inhibition of rVLM neuronal activation by the ARC and subsequent EA-related cardiovascular activation. However, direct projections from the arcuate nucleus to the rVLM, likely serve as an important source of *β*-endorphin since this projection contains this opioid peptide [41]. This latter observation is consistent with our earlier anatomical study showing that cells in the rVLM contain enkephalin but not *β*-endorphin [44]. Hence, EA-cardiovascular responses that result from the action of *β*-endorphin on *μ*-opioid receptors located on rVLM sympathoexcitatory premotor neurons depend on this hypothalamic-medullary projection [45].

## 11. Role of Spinal Cord in Acupuncture-Cardiovascular Response

The spinal cord processes somatic and visceral reflexes as well as outputs from the central nervous system to effector organs involved in cardiovascular reflex regulation [46]. Anatomical and physiological studies indicate that the dorsal horn of the spinal cord serves as a major center for EA-induced analgesia [47, 48]. Both low- and high-frequency EA at *Zusanli* (*ST 36*) acupoint increase Fos immunoreactive neurons in the superficial laminae (I and II) in the dorsal horn of the spinal cord [48]. Since opioid or nociceptin-like immunoreactivity is present in the spinal sympathetic nuclei (i.e., intermediolateral column, IML) [49, 50], we have considered the possibility that EA also influences the neurotransmission between the brain stem and the IML [51]. In this regard, our studies have found that both opioid and nociceptin reduce the response to rVLM-induced sympathoexcitation, indicating that the two peptides can regulate sympathetic outflow [52, 53]. In addition, there has been a suggestion that descending pathways from the brain stem (presumably to the dorsal horn of the spinal cord) may influence the segmental processing of somatic inputs during EA [54, 55]. Afferent stimulation can modulate sympathetic activity through the inhibition of excitatory interneurons [56]. Furthermore, somatic stimulation can elicit excitatory and inhibitory responses in both IML and dorsal horn interneurons, depending on the dermatome stimulated [57]. These interneurons appear to form important links in the spinal cord circuitry involved in autonomic control [58]. Taken together, these results indicate that opioid and nociceptin play a role in the processing of spinal cord interneuron activity in the EA response. However, spinal circuits controlling the cardiovascular visceral reflex responses during EA require further elucidation.

## 12. Endocrine and Vascular Actions of Acupuncture

Acupuncture reduces BP through modulation of the endocrine system, including decreases in plasma renin, aldosterone, and angiotensin II activity [59–61], and increased excretion of sodium [62]. Also, plasma norepinephrine, serotonin, and endorphin levels are reduced by acupuncture, reflecting its ability to modulate the neurohumoral system [63]. A laboratory-based study has demonstrated that long-term treatment with EA delayed hypertension development and restored nitric oxide in the plasma of SHRs [64]. Endothelial neuronal nitric oxide synthase (NOS) expression was significantly increased by EA in the mesenteric artery of SHRs, whereas neuronal (nNOS) expression was significantly attenuated. Additionally, EA at ST 36 induced nNOS expression in the gracile nucleus and medial nucleus tractus solitaries, and increased nNOS in the nuclei may modify central cardiovascular regulation, which contributes to hypotensive effects of acupuncture [65].

## 13. Short-Term and Long-Lasting Effect of Acupuncture

Williams and colleagues found that EA induced a significant and immediate poststimulation short-term reduction of diastolic blood pressure [66]. In 1997, a small study of 50 patients with essential hypertension found that shortly after 30 minutes of acupuncture both systolic and diastolic BP were lowered by 10–20 mmHg [61]. These data suggest that there is an immediate postacupuncture phenomenon. Our experimental studies in anesthetized animals have shown that the inhibitory effect of acupuncture on BP reflex responses occurs after 10–20 min of the start of EA stimulation and can last for as much as 60–90 min after termination of EA. In addition, in a preliminary study utilizing 24 hr ambulatory blood pressure monitoring [67], we have observed that 8 week of acupuncture lowers BP of hypertensive patients with mild-to-moderate hypertension (BP 140–180/90–110 mmHg) by 12–18 mmHg. This effect lasts for 4 weeks after termination of EA. These data suggest that EA at select acupoints (P5-P6 and ST 36-ST 37) known to have strong cardiovascular actions, performed once weekly for 8 weeks, significantly reduces BP. Importantly, this beneficial effect appears to persist for a prolonged period of time.

Several mechanisms might be involved in the long-lasting inhibitory action of acupuncture in hypertension. For example, the modulation by EA of rVLM sympathetic premotor neuronal responses to reflex-induced hypertension lasts for 30–40 min after the cessation of EA as a result of opioid and GABA modulation in this medullary region [68]. A recent study from our laboratory shows that reciprocal excitatory projections between the arcuate nucleus and the vlPAG may form a reinforcing circuit that can be activated for prolonged periods by EA, lasting as long as 30–60 min [41]. In addition, preliminary data from our laboratory using real-time PCR demonstrate that preproenkephalin in the rVLM is increased after completion of a single 30 min application of EA P 5-6 acupoints of rats [38]. The possibility that EA induces the production of opioid mRNA in the brain stem suggests that over time, EA may exert a long-lasting effect by stimulating increased production of opioid precursors.

## 14. Summary

**Figure 2:**
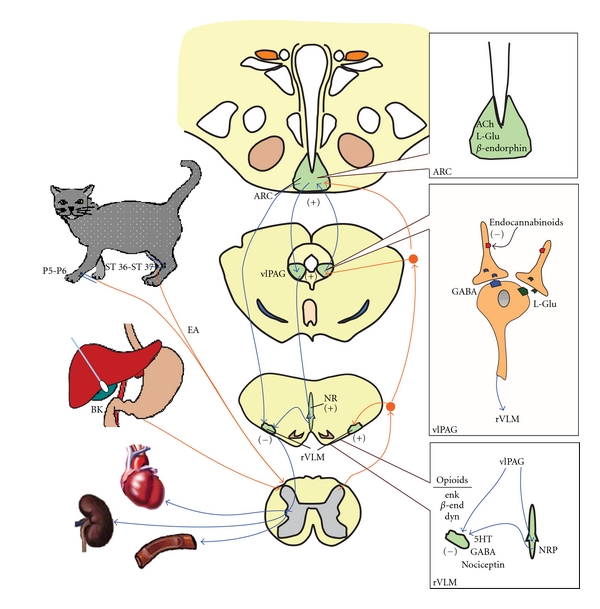
Neural circuits of acupuncture's action on cardiovascular sympathoexcitatory visceral reflex elevation of blood pressure. Abbreviations: ARC: arcuate nucleus; vlPAG: ventrolateral periaqueductal gray; NR: nuclei raphe; rVLM: rostral ventrolateral medulla. From[38].

**Figure 3:**
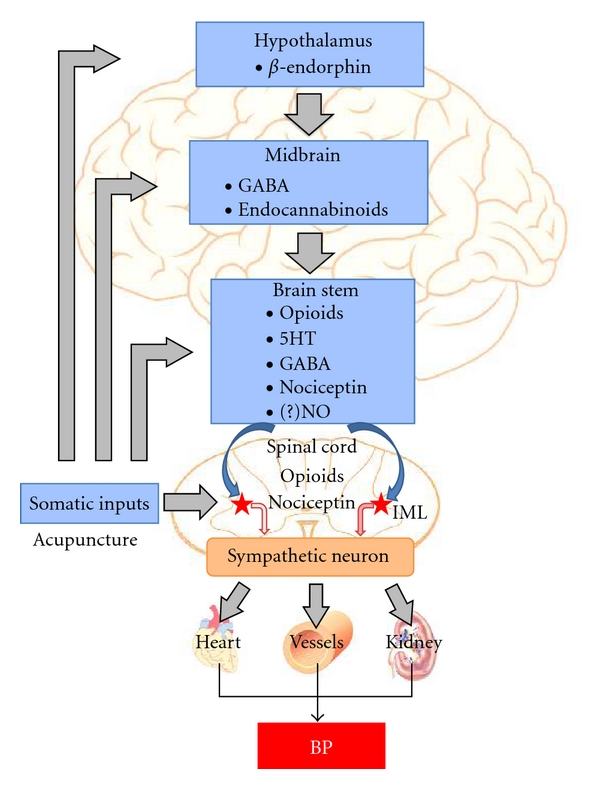
Neuroendocrine modulation of blood pressure by acupuncture. Abbreviations: GABA, *γ*-aminobutyric acid; 5HT, 5-hydroxytryptamine or serotonin; NO: nitric oxide; IML: intermediolateral column of the spinal cord.

Acupuncture has been shown to decrease BP in hypertensive patients and in animal models of hypertension. The mechanisms underlying the beneficial effects of acupuncture are associated with modulation of sympathetic outflow and possibly the endocrine system. Experimental studies have shown that EA inhibits the reflex-induced hypertension by modulating the activity of cardiovascular presympathetic neurons in the rVLM. Activation of neurons in the arcuate nucleus of the hypothalamus, vlPAG in the midbrain, and NRP in the medulla by EA can inhibit the activity of premotor sympathetic neurons in the rVLM. Glutamate, acetylcholine, opioids, GABA, nociceptin, serotonin, NO, and endocannabinoids in the brain all appear to participate in the EA antihypertensive response (Figure 2). The central action of EA may also affect the endocrine system and lead to a decrease in plasma renin, aldosterone, angiotensin II, norepinephrine, and serotonin. The neuroendocrine mechanisms of acupuncture in the treatment of hypertension are not yet fully understood, and thus are worthy of further investigation (Figure 3).

## Abbreviations

EA: Electroacupuncture
BP: Blood pressure
rVLM: Rostral ventrolateral medulla
ARC: Arcuate nucleus
vlPAG: Ventrolateral periaqueductal gray
NRP: Medullary nucleus raphé pallidus
NRO: Nucleus raphé obscurus
IML: Intermediolateral column
GABA: *γ*-aminobutyric acid
nNOS: Neuronal nitric oxide synthase
KA: Kainic acid.
